# Editorial: Improve immunity against infection and tumor by regulating gut microbiota

**DOI:** 10.3389/fcimb.2025.1546123

**Published:** 2025-05-29

**Authors:** Yue Cai, Norfilza Mohd Mokhtar, Sonia Guadalupe Sayago Ayerdi, Yongbo Kang

**Affiliations:** ^1^ Medical School, Kunming University of Science and Technology, Kunming, Yunnan, China; ^2^ Department of Infectious Disease and Hepatic Disease, The First People’s Hospital of Yunnan Province, Kunming, Yunnan, China; ^3^ Department of Physiology, Faculty of Medicine, Universiti Kebangsaan Malaysia, Kuala Lumpur, Malaysia; ^4^ Tecnologico Nacional de Mexico, Instituto Tecnologico de Tepic, Tepic, Mexico; ^5^ Department of Microbiology and Immunology, School of Basic Medical Sciences, Shanxi Medical University, Taiyuan, Shanxi, China

**Keywords:** gut microbiota, immunity, infection, cancer, probiotics

For many years, microbiota has been recognized as playing a critical role in the onset and progression of infectious diseases and cancers. In particular, the gut microbiota is known to directly impact the host immune system’s ability to fight off infection and cancer (Yoo et al., 2020). The composition and metabolites of the microbiota have been reported to regulate the maturation of the immune system, modulate the function of immune cells (e.g., CD4+ T cells), and significantly shape the immune response (Levy et al., 2016; Yongbo et al., 2022). Cancer and infection prevention and treatment strategies targeting microbiota and immunomodulation are characterized by high safety and a low risk of severe adverse effects (Jiaao et al., 2025). These approaches can be implemented through various methods, including probiotics, prebiotics, synbiotics, fecal microbiota transplantation (FMT), antibiotics, traditional Chinese medicine, and others. Preliminary research suggests that microbiota modulation may alleviate these disease (Zhu and Yang, 2025), but further mechanistic insights and more effective therapeutic strategies are still needed. This Research Topic brings together four articles that present and summarize the latest research advances that provide a better understanding of gut microbiota-based immune improvement and safe therapies for the treatment of a wide range of cancers and infectious diseases.

The role of gut microbiota in cancer therapy is widely recognized. Zhang et al. summarize the use of gut microbiota profiles to predict therapeutic response or adverse effects of chimeric antigen receptor (CAR)-T cell therapy in the treatment of hematological malignancies in order to optimize CAR-T cell therapy. The main description summarizes the current understanding of CAR-T cell therapy and the gut microbiota, as well as the interactions between the gut microbiota and CAR-T cell therapy. More importantly, potential strategies and challenges for utilizing the gut microbiota as a predictor and modulator of CAR-T cell therapy efficacy while reducing toxicity are highlighted and foreseen.

Gut microbiota is strongly associated with the development of infectious diseases. Li et al. explored the bidirectional relationship between gut microbiota and sepsis and investigated whether circulating inflammatory proteins act as mediators. The results showed 14 positive and 15 negative causal relationships between genetic susceptibility to gut microbiota and the four sepsis-related outcomes. There were causal associations between gut microbiota and circulating inflammatory proteins and all four sepsis-related outcomes. However, circulating inflammatory proteins did not appear to mediate pathways from the gut microbiota to the four sepsis-related outcomes. Notably, despite these findings, the reverse causality hypothesis does not hold. Therefore, to gain a more nuanced understanding of the relationship between gut microbiota and sepsis, future studies should focus on potential mechanistic pathways while attempting to adjust for potential confounders, such as the effects of diet, lifestyle, and medications.

Recent studies have suggested that the gut microbiota may be associated with the development of brain tumors. Yang et al. used Mendelian randomization (MR) analysis to comprehensively explore the potential causal relationship between gut microbiota and brain tumors. They found that specific intestinal microbial taxa, such as order Lactobacillus and family Clostridiaceae1, were positively associated with the development of brain tumors. In contrast, the genus Defluviitaleaceae UCG011 and the genus Flavonifracto were negatively associated with the development of brain tumors. For the first time, the study found that six gut microbiota were revealed to be associated with the risk of brain tumors, which provides a new perspective for early brain tumor prevention and treatment. These findings contribute to developing new clinical intervention strategies for preventing and treating brain tumors and point the way for future research.

Although the role of the gut microbiota in bone and joint diseases is becoming more apparent, and the concept of the “gut-joint” axis is becoming more evident, the causal relationship between the gut microbiota and septic arthritis is unclear. Bai et al. demonstrated a possible causal relationship between gut microbiota and septic arthritis by MR analysis. They found that a set of data 8 gut bacteria and 13 gut bacterial metabolic pathways were significantly and causally associated with the risk of developing septic arthritis. Among them, β-proteobacteria may be used as a marker for septic arthritis, and these findings may provide new ideas for the prevention and treatment of septic arthritis, as well as the potential for the development of novel probiotic formulations for the treatment of septic arthritis. However, future studies still need to elucidate the specific mechanisms by which these specific bacterial groups affect septic arthritis.

In summary, this Research Topic provides new ideas for diagnosing and treating infectious diseases and tumors based on the enhancement of immunity by the gut microbiota. We have learned from these efforts that advances in research have advanced the understanding of the role of the gut microbiota in tumors and infectious diseases and confirmed the causal relationship between the two. In addition, these efforts have provided some insights into precise diagnostics and therapies based on the gut microbiome and looking ahead to future directions. We look forward to more studies that elucidate the promotional or inhibitory effects of the gut microbiota on tumor and infectious disease mechanisms.

The gut microbiota plays a crucial role in shaping immune responses, offering promising strategies to combat infections and tumors. Key studies demonstrate that microbial modulation—through probiotics, prebiotics, dietary interventions, or fecal microbiota transplantation—can enhance anti-pathogen defenses and improve anti-tumor immunity by influencing immune cell function, cytokine production, and barrier integrity. These findings highlight the gut microbiome as a dynamic therapeutic target, with potential applications in immunotherapy, vaccine efficacy, and infection resistance.

However, challenges remain, including individual microbiome variability, unclear mechanistic pathways, and the need for standardized interventions. Future research should focus on: (1) Mechanistic Insights – Deciphering precise microbial-immune interactions using gnotobiotic models and multi-omics approaches; (2) Personalized Therapies – Developing microbiome-based precision medicine tailored to genetic, dietary, and microbial profiles; (3) Clinical Translation – Conducting large-scale human trials to validate efficacy, safety, and long-term outcomes of microbiota-targeted treatments; (4) Synergistic Approaches – Exploring combinations with immunotherapy, antibiotics, or immune checkpoint inhibitors to maximize therapeutic benefits.

Ultimately, harnessing the gut-immune axis represents a transformative frontier in medicine, with far-reaching implications for infectious diseases, oncology, and immune health. Continued innovation in microbiome science will be essential to unlock its full clinical potential.
